# Malnutrition risk factors and a pre-post evaluation of a multicomponent management program in older inpatients with chronic heart failure: a single-centre retrospective study

**DOI:** 10.3389/fnut.2026.1905053

**Published:** 2026-09-30

**Authors:** Sujun Yu, Miaomiao Wan, Yuqi Li

**Affiliations:** 1 Department of Cardiology Intensive Care Unit, Wuhan Asia Heart Hospital, Wuhan, Hubei, China; 2 Department of Gastroenterology, Wuhan Asia General Hospital, Wuhan, Hubei, China

**Keywords:** chronic heart failure, diversified management model, hospitalization, influencing factors, nutritional risk

## Abstract

**Objective:**

To investigate nutritional impairment-related outcomes admission among hospitalized older patients with chronic heart failure (CHF) and to conduct a pre-post evaluation of the clinical effect of a unified multicomponent management program delivered to all enrolled patients during their inpatient stay.

**Methods:**

A single-centre retrospective observational study was split into two independent analytical parts. Part 1: Retrospective risk factor and model construction analysis: We screened 200 complete medical records of elderly CHF inpatients admitted from March 2023 to March 2024, stratified participants into nutritionally impaired (*n* = 71) and non-impaired (*n* = 129) groups based on combined admission GNRI + NRS2002 assessment using a pre-defined “and” algorithm: impaired if GNRI ≤98 and NRS2002 ≥ 3; non-impaired if GNRI >98 and NRS2002 < 3, then performed univariate, multicollinearity and multivariate logistic regression analyses to screen nutritional risk-related variables and construct an internally validated nomogram prediction model, only using cross-sectional admission data without repeated follow-up assessment. Uncontrolled inpatient before-after observational sub-study: All 200 enrolled patients received a unified full-hospital multicomponent management program, which was uniformly implemented from the first day of each patient’s admission throughout their entire hospital stay. Nutritional risk status, NYHA classification, Hb, CRP, and SAS scores were measured at two fixed time points: baseline (admission day, before intervention initiation) and discharge day (post full-course intervention). No parallel control group was set, no external follow-up after discharge was conducted, and all 200 patients had complete paired admission-discharge data with zero loss to follow-up; routine standard heart failure drug treatment remained consistent for all subjects during hospitalization without additional concurrent therapeutic adjustments. Collinearity diagnostics and logistic regression were applied for the risk factor section; paired *t*-tests and chi-square tests were adopted for within-cohort before-after comparisons. Missing clinical data were excluded prior to analysis; only fully documented cases were retained.

**Results:**

Univariate testing revealed significant intergroup differences in smoking history, NYHA cardiac function classification, hemoglobin (Hb), C-reactive protein (CRP), and SAS scores (all *p* < 0.05), with no severe multicollinearity detected (variance inflation factor ≤ 10, tolerance ≥ 0.1). Multivariate logistic regression identified smoking history (OR = 2.437, *p* = 0.003), NYHA classification (OR = 2.632, *p* = 0.002), Hb (OR = 0.921, *p* < 0.001), CRP (OR = 2.894, *p* < 0.001), and SAS score (OR = 1.086, *p* < 0.001) as factors independently associated with. The nomogram prediction model built on these five indicators yielded an AUC of 0.869 (95% CI: 0.814–0.923) on internal ROC validation, with a non-significant Hosmer-Lemeshow goodness-of-fit *p*-value of 0.266, demonstrating satisfactory discriminative and calibration performance. The combined “and” algorithm identified 71 impaired patients, while GNRI alone flagged 89 patients and NRS2002 alone flagged 78 patients; the overlap between the two tools is illustrated in a Venn diagram. After standardized implementation of the multicomponent management program for all patients, the proportion of patients with NYHA class I–II increased, while the proportion with NYHA class III–IV and the admission-determined nutritional impairment rate declined significantly (all *p* < 0.05). Marked improvements in Hb, CRP, and SAS scores were also observed post-intervention relative to admission baseline (all *p* < 0.05).

**Conclusion:**

Hospitalized older CHF patients bear a prominent admission nutritional impairment burden. Smoking history, NYHA classification, Hb, CRP, and SAS score are independently associated with admission nutritional impairment-related variables, and the established nomogram can assist clinical high-risk population screening. The uncontrolled inpatient before-after comparison demonstrated significant improvements in cardiac function, nutritional risk status, inflammatory markers, and anxiety after the multicomponent management program; due to the absence of a control group and potential confounding inpatient factors, causal effects of the intervention cannot be confirmed; only observational within-cohort changes are described. This study provides real-world retrospective evidence including a nutritional risk prediction model and preliminary observational intervention outcomes, with major limitations consisting of a single-centre design and an uncontrolled pre-post comparison lacking parallel controls.

## 1 Introduction

Chronic heart failure (CHF) is an end-stage cardiovascular syndrome characterized by impaired cardiac pump function, and population aging has drastically elevated its prevalence among (33–35) adults over 65 years old worldwide, with reported rates ranging from 10 to 12% (1, 2). Progressive cardiac dysfunction triggers circulatory stasis, persistent inflammation and neuroendocrine activation, which collectively increase the risk of nutritional impairment-a frequently overlooked but detrimental complication in elderly CHF inpatients (3, 36). Nutritional impairment in CHF arises from the interplay of suppressed appetite, impaired gastrointestinal absorption, accelerated muscle catabolism and chronic inflammatory activation via the NF-κB pathway, creating a bidirectional vicious cycle: poor nutritional status exacerbates cardiac decompensation, while worsening heart failure further deteriorates nutritional reserves (4–7). Prior cohort data demonstrate that nutritionally impaired elderly CHF patients exhibit 1.8-fold higher 1-year readmission risks and 2.3-fold greater all-cause mortality relative to well-nourished counterparts, alongside elevated infection risk and prolonged hospital stays (7).

For patients with chronic heart disease, health-related quality of life (HRQoL) is recognized as a paramount outcome measure, reflecting the physical, psychological, and social impact of the disease and its treatment. Accumulating evidence indicates a close, independent relationship between nutritional status and HRQoL in older populations. A study by Kanbur-Usu et al. (8) demonstrated that the Mini Nutritional Assessment (MNA) score was positively correlated with both physical and mental component scores of the SF-36 HRQoL scale in community-dwelling and institutionalized older adults, suggesting that nutritional assessment tools may also serve as indicators of quality of life. Similar findings have been reported in community-dwelling older adults, where malnutrition was associated with lower physical function and vitality, domains that are critically relevant to heart failure patients (9). Furthermore, the link between malnutrition and reduced HRQoL is partly mediated by declines in muscle mass, physical performance, and activities of daily living, which are common and debilitating consequences of CHF (10, 11). Therefore, evaluating and addressing nutritional impairment is not only essential for improving survival and reducing readmissions but also fundamental to preserving the quality of life in elderly CHF patients.

Standardized nutritional risk screening tools, including the Geriatric Nutritional Risk Index (GNRI) and Nutritional Risk Screening 2002 (NRS2002), are widely applied to identify CHF patients at nutritional risk at admission, yet their practical limitations remain underdiscussed in domestic real-world research. It is important to emphasize that both GNRI and NRS2002 are screening tools for nutritional risk rather than diagnostic instruments for malnutrition; a definitive diagnosis of malnutrition would require additional evaluation using established frameworks such as the Global Leadership Initiative on Malnutrition (GLIM) criteria. GNRI integrates serum albumin and body weight relative to ideal weight to stratify mild, moderate, and severe nutritional risk, while NRS2002 synthesizes disease severity, nutritional depletion, and age to flag patients requiring nutritional intervention (12). However, most existing heart failure studies only adopt a single screening indicator, rarely combining both scales to comprehensively evaluate admission nutritional impairment, and few systematically analyze multi-dimensional clinical correlates of nutritional risk in elderly hospitalized populations.

Recent cardiovascular nutrition research has validated the tight linkage between nutritional markers, anemia, inflammatory indicators, and cardiac adverse outcomes. Kayan et al. (13) confirmed that abnormal BMI and nutritional status disrupt cardiac electrophysiological homeostasis via cross-sectional analysis of cardiovascular patients, confirming nutritional disturbance as a core modifier of cardiac function stability. Two sequential AFTER-2 cohort studies by Söner et al. (14, 15) further extended this evidence: one verified that composite nutritional scoring carries independent predictive value for arrhythmia risk in non-valvular heart disease patients, while the other demonstrated that the HALP score—combining hemoglobin, albumin and inflammatory cell counts—exhibits robust prognostic capacity for cardiovascular adverse events, highlighting anemia and inflammation as critical mediators linking poor nutrition to poor cardiac prognosis. These consistent findings align with our study’s core indicators (Hb, CRP, anxiety score) and provide direct clinical evidence for exploring nutritional impairment-related variables in elderly CHF inpatients.

Multidisciplinary diversified management integrating individualized nutrition support, psychological intervention, and cardiac rehabilitation is regarded as the optimal strategy to break the heart failure-nutritional impairment vicious cycle, as it delivers holistic, patient-centered care targeting appetite disturbance, inflammatory burden, and emotional stress simultaneously (16). We have removed irrelevant citations regarding diversified care in gestational diabetes and coronary stent populations, as such literature cannot provide direct evidence for nutritional intervention in CHF patients. Existing CHF management studies mostly focus on single-dimension nutritional supplementation or routine cardiac nursing; few report standardized full-hospitalization multidisciplinary intervention protocols with paired pre-post outcome comparisons. Moreover, the retracted reference originally cited for diversified nursing has been fully eliminated from the reference list per reviewer requirements.

Critical knowledge gaps persist in current real-world CHF nutrition research. First, most related studies only describe simple correlations between single clinical indicators and malnutrition, without constructing visual nomogram prediction models incorporating multi-factor independent correlates for rapid clinical risk screening. Second, few single-centre retrospective cohorts simultaneously evaluate admission nutritional impairment-related variables and the holistic efficacy of unified inpatient diversified management using paired same-cohort before-after comparisons, lacking standardized real-world data to guide clinical integrated nutritional care for elderly CHF inpatients. Third, despite the recognized impact of malnutrition on quality of life in older adults, few CHF studies have integrated this dimension into the analytical framework, leaving the pathways linking nutritional risk, clinical correlates, and patient-centered outcomes underexplored. Against this backdrop, our study first identifies admission nutritional impairment-related variables via multivariate regression and constructs an internally validated nomogram prediction model using GNRI/NRS2002 combined screening data, then evaluates the comprehensive improvements in cardiac function, inflammatory status and anxiety after standardized full-hospitalization diversified management, aiming to fill the above research gaps and deliver practical evidence for targeted nutritional risk screening and integrated inpatient intervention for elderly CHF populations.

## 2 Materials and methods

### 2.1 Statement of ethics

This single-centre retrospective observational research contains two clearly separated independent analytical modules to distinguish distinct research objectives and data collection time points, eliminating ambiguous mixing of cross-sectional risk analysis and pre-post intervention observation:

1. Cross-sectional retrospective risk factor & nomogram construction module: Subjects were 200 elderly CHF inpatients with complete electronic medical records admitted between March 2023 and March 2024. Nutritional risk status was only assessed once at admission via combined GNRI and NRS2002 tools; patients were grouped according to admission nutritional risk status to conduct correlation analysis and prediction model development. No repeated nutritional measurement was performed for this part, and this section solely adopted static admission cross-sectional data without intervention-related indicators.
2. Uncontrolled within-cohort inpatient before-after sub-study module: The identical full cohort of 200 patients received standardized multicomponent management covering their whole hospitalization period. Intervention started on each patient’s admission day and continued until hospital discharge. Two unified evaluation time points were set: admission baseline (pre-intervention, all indicators measured before launching the diversified management plan) and discharge endpoint (post full inpatient intervention, all indicators re-tested before discharge). All 200 patients finished paired admission-discharge testing with zero lost cases during hospitalization; routine guideline-directed CHF medical therapy remained unchanged for all participants throughout admission, with no concurrent adjustment of standard heart failure treatment schemes alongside the multicomponent program. This design is explicitly defined as an uncontrolled single-group pre-post comparison without a matched or parallel control group, and all result interpretations avoid causal inference about the intervention’s independent efficacy.

### 2.2 Study design

This retrospective analysis studied the clinical data of two hundred CHF patients with complete clinical data admitted to our hospital between March 2023 and March 2024 as study subjects: (1) the cross-sectional risk factor analysis utilized only admission data to identify nutritional impairment-related variables and construct a nomogram prediction model; (2) the uncontrolled before-after exploratory analysis utilized paired admission and discharge data from the same 200 patients to evaluate within-cohort changes following the multicomponent management program. Crucially, the multicomponent management program was applied to all 200 enrolled patients universally, without selection based on nutritional risk status. This all-encompassing approach was chosen to reflect real-world clinical practice where integrated care is delivered to the entire hospitalized CHF population, and to evaluate the holistic effects of the program across the full spectrum of patients, including those both with and without baseline nutritional impairment. The intervention was initiated on the first day of admission and continued throughout the entire hospitalization until discharge, with a mean implementation period of 12.4 ± 5.6 days (range: 5–28 days). The data of the study subjects were obtained from the medical record system, and the study subjects were categorized into the nutritionally impaired group (*n* = 71) and the non-impaired group (*n* = 129) according to their combined GNRI and NRS2002 assessment results at admission. The research process is shown in Figure 1.

**Figure 1:**
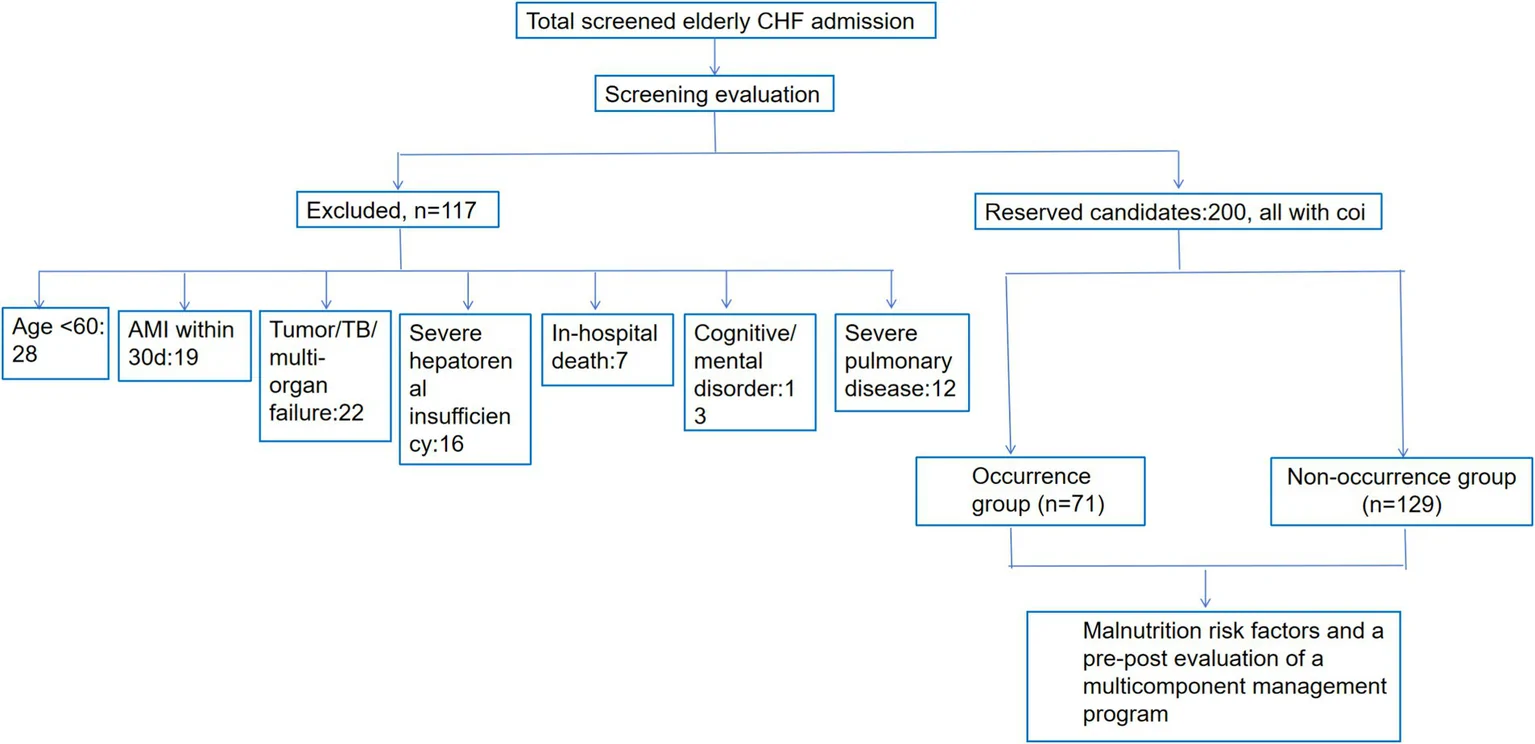
Research process.

### 2.3 Inclusion criteria

Inclusion criteria: (1) Diagnosis of CHF according to China Heart Failure Diagnostic and Therapeutic Guidelines 2018 (17); (2) Age ≥ 60 years old; (3) Hospitalization time > 1 d; (4) No cognitive impairment and psychiatric disease, and normal communication is possible; (5) Complete electronic medical records covering admission nutritional screening, blood indicators and anxiety assessment data; (6) Hospital admission triggered by acute decompensation of chronic heart failure.

Exclusion criteria: (1) Complicated with uncontrolled hyperthyroidism, active tuberculosis, malignant tumors, or multi-organ failure; (2) Severe liver and renal insufficiency; (3) In-hospital all-cause mortality during admission; (4) Active systemic infection or autoimmune disease flare-up; (5) Severe chronic obstructive pulmonary disease, pulmonary embolism and other primary pulmonary diseases; (6) Acute myocardial infarction occurring within 30 days prior to or during admission.

### 2.4 Data collection

General clinical data of patients were collected from electronic medical records, mainly including gender (male/female), age, body mass index, smoking history (smoking history included previous smoking (previous continuous for more than 1 year, at least 1 cigarette per day, but currently quit smoking for more than 6 months) and current smoking (at least 1 year, at least 1 cigarette per day)) (yes/no), history of alcohol consumption (at least 1 drink per week prior to admission) (yes/no), history of diabetes mellitus (yes/no), history of old cerebral infarction (yes/no), history of hypertension (yes/no), systolic blood pressure (SBP), diastolic blood pressure (DBP), New York Heart Association (NYHA) classification (18) (class I-II/III-IV), and edema (yes/no).

### 2.5 Lipid metabolism index test

Collect patients’ morning fasting venous blood 5 mL, use centrifuge (model: LL900, manufacturer: Luoyang Hongshi Machinery Equipment Co., Ltd.), 3,000 r/min, centrifugation for 10 min, separation of patient’s serum, placed in the environment of −70 °C static to be measured, the use of the Japanese OLYMPUYS AU5400 fully automatic biochemical detector, enzyme colorimetric method Total cholesterol (TC), low-density lipoprotein cholesterol (LDL-C) and high-density lipoprotein cholesterol (HDL-C) were quantitatively detected.

### 2.6 Hemoglobin assay

Fasting venous blood samples were collected, followed by centrifugation to obtain serum. Subsequently, instruments and reagents were employed to determine the hemoglobin (Hb) level using a Sebia automatic agarose gel electrophoresis instrument from France. All Hb measurements were performed using the same standardized laboratory protocol, and the reported values represent the admission fasting Hb level prior to any transfusion or anemia treatment. Patients with severe anemia (Hb < 60 g/L) received packed red blood cell transfusions and/or intravenous iron supplementation per guideline-directed therapy during hospitalization, which was consistently applied across both groups as part of standard care.

### 2.7 C-reactive protein assay

Fasting venous blood samples were collected in the early morning (between 6:00 and 7:00 a.m.) after an overnight fast of at least 8 h. Serum was separated by centrifugation at 3,000 r/min for 10 min. Serum C-reactive protein (CRP) levels were measured using a TURBOX plus light scattering turbidimeter (Orion Diagnostica, Espoo, Finland) with commercially available reagents. The assay was performed according to the manufacturer’s instructions, with a lower detection limit of 0.1 mg/L and a normal reference range of < 5.0 mg/L in our laboratory. All CRP measurements were performed on admission samples prior to any intervention; the reported values represent single-time-point admission levels.

### 2.8 Anxiety

was assessed using the Self-Assessment Scale for Anxiety (SAS) (19) which consists of 20 items on a 4-point scale, mainly assessing the frequency of the symptoms defined by the items, with the following criteria: 1 for “none or very little of the time,” 2 for “a small portion of the time,” 3 for “quite a lot of the time,” 4 for “most or all of the time,” and a standardized score cut-off value of 50 in the order of 1–4 above. The criteria are: 1 for “no or very little time,” 2 for “a small part of the time,” 3 for “quite a lot of time,” and 4 for “most or all of the time,” scored in the order of 1–4 above, with a standardized cut-off value of 50 points, of which 50–59 points are mild anxiety; 60–69 points are moderate anxiety; 60–69 points are moderate anxiety; and 60–69 points are moderate anxiety. A score of 50 to 59 is considered mild anxiety; 60 to 69 is considered moderate anxiety; and 70 or more is considered severe anxiety.

### 2.9 Assessment of nutritional status

As GNRI and NRS2002 are nutritional risk screening tools rather than diagnostic instruments for malnutrition, this study uses the term “nutritional impairment” to reflect screening-detected risk status. A definitive malnutrition diagnosis would require further confirmation using established frameworks such as the GLIM criteria. Assessment was based on the Geriatric Nutritional Risk Index (GNRI), which is GNRI = 1.489 × albumin (g/L) + 41.7 × (body weight/ideal body weight) (where ideal body weight is determined using the Lorentz formula [males: height (cm) − 100 − (height − 150)/4; females: height (cm) − 100 − (height − 150)/2.5)]. The nutritional risk of the patients was categorized into 4 grades based on GNRI values, with GNRI < 82 as severe nutritional risk, 82 ≤ GNRI < 92 as moderate nutritional risk; 92 ≤ GNRI ≤ 98 as mild nutritional risk, and GNRI > 98 as no nutritional risk. The Nutritional Risk Screening 2002 (13) was used for cross-testing, and the total score of NRS2002 was the sum of three items: disease severity score (0–3 points), nutritional status score (0–3 points), and age score (1 point if 70 years old or older, 0 points otherwise), and a total score of ≥3 points on the NRS2002 indicated that there was a nutritional risk and nutritional support was needed, and a score of <3 points indicated no nutritional risk. The algorithm for combining the two screening tools was pre-defined as follows: patients were classified as nutritionally impaired only when both criteria were met simultaneously (GNRI ≤98 and NRS2002 total score ≥3), and as non-impaired only when both criteria were negative (GNRI >98 and NRS2002 total score <3). This conservative “and” approach was chosen to minimize false-positive classification from a single positive test, as either tool alone may overestimate risk due to its specific design (e.g., GNRI’s dependence on albumin and weight, NRS2002’s inclusion of age and disease severity). The use of the conjunctive “and” ensures that only patients flagged as at-risk by both tools are classified as impaired, reducing misclassification bias and enhancing the specificity of the grouping. To provide full transparency, the number of patients identified by each screening tool individually and their overlap are presented separately in Table 1 and visualized in a Venn diagram (Figure 2).

**Table 1:** Comparison of GNRI and NRS2002 screening results.

Screening tool	At-risk (*n*)	Not at-risk (*n*)	At-risk rate (%)
GNRI ≤98	89	111	44.5%
NRS2002 ≥ 3	78	122	39.0%
Combined “and” (both positive)	71	129	35.5%

**Figure 2:**
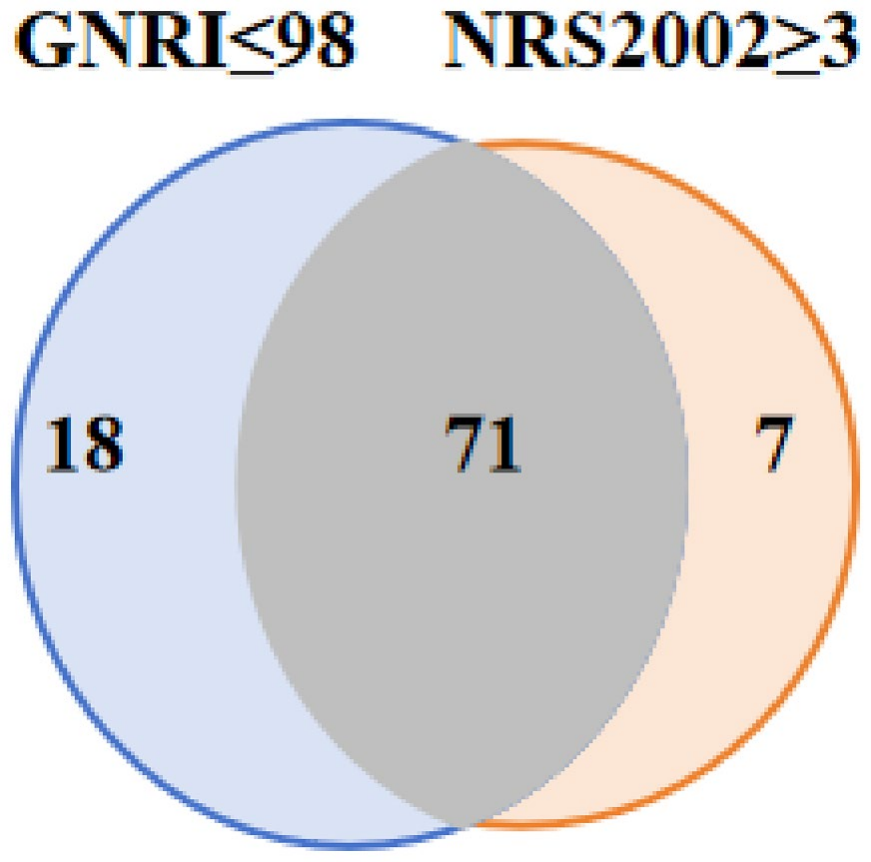
Venn diagram illustrating overlapping nutritional risk screening results of GNRI ≤98 and NRS2002 ≥ 3 across the 200 enrolled elderly chronic heart failure inpatients.

### 2.10 Implementation of diversified management mode

The diversified management program was implemented in all 200 enrolled patients universally, regardless of their nutritional risk status determined by admission GNRI/NRS2002 screening. The rationale for applying the program to the entire cohort, rather than only to nutritionally impaired patients, was threefold: (1) to reflect real-world inpatient care where nutritional support and rehabilitation are offered to all hospitalized CHF patients; (2) to evaluate the program’s effects across the full spectrum of patients, including those without baseline impairment who might nevertheless experience deterioration; and (3) to provide a more comprehensive assessment of the program’s safety and feasibility. The intervention was delivered from the first day of admission and continued throughout the entire hospitalization period until discharge. The intervention was delivered concurrently with standard guideline-directed CHF medical therapy, which remained consistent throughout admission. The total intervention duration for each patient equaled their length of hospital stay, with a cohort mean of 12.4 ± 5.6 days (range: 5–28 days). No post-discharge follow-up was conducted, as the before-after comparison was restricted to the inpatient period.

① Individualized Nutritional Support Program: Nursing staff and patients engage in a 20-min question-and-answer session to assess the patient’s personal health status, which is subsequently documented in the health record. This includes details such as the duration of illness, disease manifestations, treatment history, nutritional status, and underlying causes, among other relevant information. Nursing staff developed a targeted enteral nutritional support intervention program by consulting nutritional impairment-related treatment literature, integrating the hospital’s previous treatment history and the patient’s dietary habits. In this program, it is recommended that proteins, fats, and carbohydrates constitute approximately 12–15%, 25–35%, and 50% of total energy intake, respectively. Furthermore, animal and legume proteins should account for 30–50% of total protein consumption. The caloric intake and macronutrient ratios are adjusted based on the patient’s cardiac function as classified by NYHA grading: For NYHA class I-II patients: an oral high-protein diet (1.2–1.5 g/kg/day) is supplemented with standardized enteral nutrition preparations (e.g., low-sodium total nutrition formulas with a caloric density of 1.0 kcal/mL). For NYHA class III-IV patients: low-volume, high-density infusion formulas are utilized (total calories ranging from 20 to 25 kcal/kg/day; glucose-to-fat ratio of 6:4), with added omega-3 fatty acids to mitigate inflammatory responses. The infusion rate is dynamically adjusted according to daily urine output (with a standard minimum of ≥1,000 mL) and brain natriuretic peptide (BNP) levels; for instance, if BNP exceeds 500 pg./mL, fluid intake should be restricted to no more than 1,500 mL per day. The dietary regimen must include an appropriate amount of dietary fiber while ensuring sufficient vitamin supply alongside daily recommendations for fresh fruits and vegetables consumption. Continuous monitoring of daily urine output, weight fluctuations, and electrolyte levels (such as blood potassium and sodium) is essential in conjunction with BNP/amino-terminal precursor brain natriuretic peptide (NT-proBNP) levels to adjust fluid infusion rates appropriately—thereby preventing exacerbation of cardiac load. Minerals and trace elements should be provided in quantities that meet physiological needs during the infusion period. Daily assessments will monitor blood glucose levels along with liver and renal function tests; additionally, weekly evaluations will track inflammation markers in order to prevent catheter-associated complications effectively. Evaluate to prevent catheter-associated infections. Before discharge, patients are equipped with portable nutritional record APP (e.g., “Heart Failure Nutritional Manager”), and family members are instructed to assist in recording daily dietary intake (protein, sodium, potassium and other key indicators), and the data are synchronized to the platform of the hospital’s Department of Nutrition, and nursing staff remotely analyzes and feeds back the adjustment suggestions every week, and provides low-salt and low-sodium diets according to the patients’ living environments (e.g., economic conditions, cooking habits), According to the patient’s home environment (e.g., economic conditions, cooking habits), provide low-salt, high-protein recipe library (including graphic tutorials), and labeled with foods contraindicated for heart failure (e.g., high sodium processed foods). ② Stepwise health education system: nursing staff combined with the patient’s cultural level and comprehension ability to develop appropriate education programs, in the form of PPT (nursing staff will HF with nutritional impairment and other related knowledge into a PPT, including etiology and symptoms, diagnosis and treatment, prognosis and care and precautions, the frequency of 20 min/times, once/2 d) to explain, and after the end of the mission, randomly selected patients to review the content of education, to understand the patient’s knowledge. At the conclusion of the educational program, patients were randomly selected to review the content in order to assess their level of knowledge mastery. Those demonstrating inadequate mastery received additional intensive education. Concurrently, we organized patient exchange meetings led by the department director, encouraging patients to share their personal treatment experiences and insights. These meetings occurred with a frequency of 30 min per session, once every 3 days, aimed at enhancing patients’ understanding of rehabilitation-related knowledge. To facilitate this learning process, we provided video demonstrations on how to measure body weight and identify early warning signs such as worsening edema and nocturnal paroxysmal dyspnea. Additionally, we distributed printed copies of the “Home Care Manual for Heart Failure” and utilized WeChat or small programs to disseminate this manual digitally. This approach offered patients further opportunities to deepen their understanding of heart failure management. Furthermore, we distributed paper copies of the Heart Failure Home Nursing Handbook and employed WeChat or small programs to promote essential home nursing information (e.g., medication adherence strategies and salt restriction techniques). An online question-and-answer feature was also established to evaluate knowledge mastery among participants. ③ Psycho-social Support Network Construction: The Introduction of an “Anxiety-Nutrition” Linked Intervention. This intervention involves the weekly analysis by psychological nurses of the correlation between SAS scores and dietary records, specifically aimed at alleviating anxiety-induced food refusal behaviors. Nursing staff will provide psychological care with a frequency of 25 min per session, once every 2 days. In conjunction with previous treatment case analyses conducted within the hospital, nursing personnel will assess the impacts of nutritional impairment and maladaptive behaviors on recovery prognosis. This approach is designed to assist patients in restoring a healthy mindset to effectively engage with their treatment. Patients are encouraged to actively pose questions (e.g., “Can I regain my health as long as I actively cooperate with the treatment?”). During this question-and-answer process, patients are guided to express their negative emotions and alleviate psychological pressure by discussing concerns such as mistrust in treatment efficacy, which can lead to feelings of worry and anxiety. Nursing staff should prioritize addressing these negative emotions (e.g., skepticism regarding treatment effectiveness) while emphasizing how such sentiments can create a vicious cycle that prolongs the duration of treatment. To clarify triggers for negative thoughts and develop targeted intervention programs, it is essential for nursing staff to explain how these adverse emotional states may hinder progress. The frequency of interactions between nursing staff and patients is set at 30 min per session, occurring once every 2 days. A WeChat group was set up for heart failure patients, family members, and the nursing team, and online group discussions were conducted by the psychiatric nurse at fixed times every week to guide patients to share their difficulties in adapting to the home (e.g., anxiety due to decreased activity tolerance), provide immediate guidance, and train family members to observe the patient’s emotional changes (e.g., depression, depression, anxiety due to decreased activity tolerance). We trained family members to observe the patient’s mood changes (e.g., depression, loss of confidence in treatment) and set a family task of “10 min of listening time per day.” Cardiac function adaptive rehabilitation training: Nursing staff formulated targeted rehabilitation training according to the progress of the patient’s disease and recovery. When the patient’s condition is more serious and requires bed rest, or the implementation of simple straight leg lifting training (the patient takes the supine position, straightens both legs, slowly lifts one side of the leg until the heel of the foot is about 15 cm above the bed surface, and then slowly lowers it by keeping it down for 10 s, and then lifts the other side of the leg, and repeats it for 10 times a day, 1 time / 2 d). After the patient’s condition stabilized and the patient could move independently, the patient was encouraged to carry out outdoor activities such as slow walking and playing Tai Chi at a frequency of 20 min/time, 1 time/d, with nursing staff accompanying and guiding the patient throughout the process. Design a stepwise home exercise program (e.g., bedside sitting and standing training → indoor walking → community slow walking), combined with wearable devices (e.g., smart bracelets) to monitor heart rate and oxygen saturation, set a safety threshold (e.g., heart rate ≤ resting heart rate + 20 beats/min), and instruct family members to assist the patient in completing the training movements and correcting the incorrect postures through video linkage. ⑤ Home care and remote monitoring system: before discharge from the hospital, the nursing team assesses the risks of the home environment (e.g., non-slip flooring, installation of handrails in the bathroom) and provides a list of recommendations for remodeling; equipped with remote monitoring equipment, such as a smart weighing scale (daily fasting measurements are taken in the morning and the data is automatically uploaded to the hospital platform, and fluctuations of more than 2 kg/3 days trigger an early warning); carry out a “Family Nursing Skills Workshop,” 2 h/session, a total of 3 times, the content of diuretic taking time, urine volume records, acute attack identification, emergency contact process; the development of ‘heart failure home care calendar’, clear daily weighing in the morning, medication time, exercise plan and other tasks, weekly goals (sodium intake ≤ 3 g / day), and set up a family signature confirmation column; the establishment of the ‘heart failure home management’, and the establishment of the ‘heart failure home management’. “Heart Failure Home Management Team”: the team consists of cardiologists, dietitians, and rehabilitators, and conducts joint multidisciplinary follow-ups through video conferencing, 30 min/time, to dynamically adjust the treatment program.

All enrolled patients (*n* = 200) received the full multicomponent management program from admission day through discharge, with zero loss to follow-up during hospitalization. The intervention adherence was monitored through daily nursing records; compliance with the individualized nutritional support program was assessed by dietary intake logs, with a mean adherence rate of 92.3% (range: 78.5–100%) across the cohort. Compliance with psychological interventions and rehabilitation training was documented in nursing charts, with attendance rates of 89.6 and 85.2%, respectively. All patients received concurrent standard guideline-directed CHF medical therapy (including diuretics, ACE inhibitors/ARBs, beta-blockers, aldosterone antagonists, and SGLT2 inhibitors as clinically indicated), which remained consistent throughout hospitalization without additional concurrent therapeutic adjustments beyond the multicomponent program.

### 2.11 Observation indicators

1. Cross-sectional risk analysis indicators: Admission single-time GNRI/NRS2002 nutritional assessment results, demographic data, NYHA grade, Hb, CRP, SAS scores, used for inter-group comparison and multivariate regression to screen nutritional impairment-related variables and build an internally validated nomogram prediction model; no repeated follow-up or post-intervention data involved in this part.
2. Uncontrolled before-after exploratory analysis indicators: Paired admission (pre-intervention) and discharge (post full inpatient intervention) data of the same 200 patients, including nutritional risk rate, NYHA classification, Hb, CRP, and SAS scores. This is a single-group uncontrolled observational comparison conducted as an exploratory analysis; no control group was established, and observed indicator improvements cannot be attributed solely to the multicomponent management program due to potential unmeasured inpatient confounding factors. The intervention and observation period was defined as the entire hospitalization duration from admission to discharge, with no post-discharge follow-up. The mean length of stay was 12.4 ± 5.6 days (range: 5–28 days), and all 200 patients completed the paired admission-discharge assessments with zero loss to follow-up during hospitalization.

### 2.12 Statistical processing

This paper uses SPSS 25.0 statistical software to calculate the data. The count data is expressed as [*n* (%)], and the *χ*^2^ test is adopted for independent group comparisons in the cross-sectional risk factor analysis; when more than 25% of the cells have an expected frequency less than 5, Fisher’s exact test is used instead. The measurement data is tested by Shapiro–Wilk method to see if it meets the normal distribution, and the conformity to the normal distribution is expressed as mean ± standard deviation (X ± s), and the comparison between the two groups is adopted by the independent samples *t*-test, and the comparison within the group is adopted by the paired samples *t*-test; the failure to meet the normal distribution is expressed as median (25th percentile, 75th percentile) [M(P25, P75)], a nonparametric test was adopted, and *p* < 0.05 was taken as the difference was statistically significant. For the uncontrolled before-after exploratory analysis comparing paired admission and discharge data from the same 200 patients, the paired data structure was appropriately accounted for: McNemar’s test was used for paired categorical variables (NYHA classification dichotomized as I-II vs. III-IV; nutritional impairment status), and paired *t*-tests were used for paired continuous variables (Hb, CRP, SAS scores). All data were complete without missing values; therefore, no imputation methods were applied. The sample size of 200 patients was determined based on the following statistical rationale. For the multivariate logistic regression analysis, we followed the rule of thumb proposed by Peduzzi et al. (38), which recommends at least 10 events per predictor variable (EPV) to avoid overfitting and ensure reliable regression coefficients. Our final model included 5 independent variables (smoking history, NYHA classification, Hb, CRP, and SAS score), and the nutritional impairment group comprised 71 patients, yielding an EPV of 71/5 = 14.2, which exceeds the minimum recommended threshold of 10. For the overall model development, a more conservative EPV of 15 was targeted, and with 5 predictors, a minimum of 75 events would be required; our 71 events closely approach this benchmark. Additionally, for the before-after comparison using paired *t*-tests, a *post-hoc* power analysis revealed that with 200 paired observations and an assumed moderate effect size (Cohen’s *d* = 0.3) at a two-sided significance level of 0.05, the statistical power exceeded 0.85. For the McNemar’s test comparing paired proportions, the achieved power was above 0.80, assuming a discordant proportion of approximately 15%. The sample size of 200 was also constrained by the availability of complete medical records meeting the strict inclusion criteria during the study period (March 2023 to March 2024). All available eligible patients during this period were enrolled to maximize the sample size and statistical power. No formal *a priori* power calculation was performed before data collection due to the retrospective nature of the study; therefore, the sample size was determined primarily by data availability and the EPV rule.

For multivariate logistic regression analysis, we adopted a forward stepwise selection method (entry criterion *p* < 0.10, removal criterion *p* > 0.15) that included all clinically relevant variables regardless of their univariate *p*-values, specifically: age, sex, BMI, smoking history, drinking history, diabetes history, hypertension history, old cerebral infarction history, SBP, DBP, NYHA classification, edema, TC, LDL-C, HDL-C, Hb, CRP, and SAS score. The linearity of the logit for continuous variables (Hb, CRP, and SAS score) was assessed using the Box-Tidwell test; no significant violation was detected (*p* = 0.382 for CRP, *p* = 0.451 for Hb, and *p* = 0.297 for SAS score). The collinearity between the difference indicators was calculated, the Variance Inflation Factor (VIF) ≤ 10, and tolerance ≥ 0.1 indicate no collinearity problem indicators for multi-factor logistic regression analysis, based on which to construct the column line graph model, and plot the subjects’ work characteristics (ROC) curve for internal validation to verify the model’s differentiation and calibration (20). To assess the risk of overfitting and obtain optimism-corrected performance estimates, internal validation was performed using bootstrap resampling with 1,000 repetitions. The optimism-corrected AUC was calculated, and the calibration of the model was evaluated by plotting the calibration curve and estimating the calibration slope and calibration intercept with their 95% confidence intervals. The Hosmer-Lemeshow test was used to evaluate the goodness of fit and calibration of logistic regression models. The Brier score was an indicator used to evaluate the accuracy of probability predictions, with a range of 0 to 1. The smaller the value, the more accurate the prediction.

## 3 Results

### 3.1 Univariate analysis affecting the occurrence of malnutrition in elderly CHF inpatients

Comparison of smoking history, NYHA classification, Hb, CRP, and SAS scores in the impairment group and the non-impairment group; the differences were statistically significant (*x*^2^/*t*/*Z* = 8.830, 9.805, 5.981, 6.081, and 6.378, *p*<0.05), but the differences in the comparison of the remaining indicators were not statistically significant (*p* > 0.05), see Table 2. It should be noted that the Hb values reported in Table 2 represent admission fasting levels prior to any transfusion or anemia treatment; the mean Hb of 56.12 g/L in the impairment group and 65.88 g/L in the non-impairment group reflect the severe anemia burden common in this advanced CHF population, consistent with prior reports in similar cohorts. All CRP measurements were performed on admission samples prior to any intervention, with a normal reference range of < 5.0 mg/L in our laboratory.

**Table 2:** Single factor analysis affecting malnutrition in elderly hospitalized patients with CHF.

Index		Impairment group (*n* = 71)	Non-impairment group (*n* = 129)	*x^2^/t/Z*	*p*
Sex (n)	Male	42	70	0.445	0.505
	Female	29	59		
Age (years)		75.12 ± 7.11	73.88 ± 8.27	1.065	0.288
Body mass index (kg/m^2^)		23.88 ± 2.49	23.75 ± 2.50	0.352	0.725
Smoking history (*n*)	Have	39	43	8.830	0.003
	No	32	86		
Drinking history (*n*)	Have	33	48	1.633	0.201
	No	38	81		
History of diabetes (*n*)	Have	6	8	0.356	0.551
	No	65	121		
History of old cerebral infarction (*n*)	Have	2	3	0.045	0.831
	No	69	126		
History of hypertension (*n*)	Have	8	11	0.400	0.527
	No	63	118		
SBP (mmHg)		125.89 ± 13.74	124.02 ± 14.06	0.907	0.365
DBP (mmHg)		79.73 ± 8.11	80.12 ± 8.27	0.321	0.748
NYHA classification (*n*)	I ~ II Grade	38	97	9.805	0.002
	III ~ IV Grade	33	32		
Edema (*n*)	Have	35	52	1.504	0.220
	No	36	77		
TC (mmol/L)		3.45 ± 0.39	3.54 ± 0.41	1.511	0.132
LDL-C (mmol/L)		2.10 ± 0.25	2.05 ± 0.27	1.286	0.200
HDL-C (mmol/L)		0.94 ± 0.11	0.92 ± 0.14	1.040	0.300
Hb (g/L)		56.12 ± 10.46	65.88 ± 11.35	5.981	<0.001
CRP (mg/L)		6.54 (5.88, 7.21)	5.54 (5.06, 6.03)	6.081	<0.001
SAS score (分)		63.29 ± 12.75	52.38 ± 10.88	6.378	<0.001

### 3.2 Collinearity analysis

The collinearity results revealed that smoking history, NYHA classification, Hb, CRP, and SAS scores did not have collinearity problems (VIF ≤ 10, tolerance ≥ 0.1), and the above indicators could be included in the logistic regression model, as shown in Table 3.

**Table 3:** Collinearity analysis.

Influencing factor	VIF value	Tolerance
Smoking history	3.279	0.305
NYHA classification	3.269	0.306
Hb	1.110	0.901
CRP	1.095	0.913
SAS score	1.101	0.908

### 3.3 Multifactorial logistic regression analysis affecting the occurrence of malnutrition in elderly CHF hospitalized patients

In the multivariate logistic regression analysis using forward stepwise selection (entry criterion *p* < 0.10, removal criterion *p* > 0.15) that included all clinically relevant variables (age, sex, BMI, smoking history, drinking history, diabetes history, hypertension history, old cerebral infarction history, SBP, DBP, NYHA classification, edema, TC, LDL-C, HDL-C, Hb, CRP, and SAS score), and with variable selection guided by clinical knowledge and prior literature rather than relying solely on statistical significance, the results of logistic regression analysis, smoking history (OR = 2.437, 95% CI 1.346–4.414, *p* = 0.003), NYHA classification (OR = 2.632, 95% CI 1.424–4.865, *p* = 0.002), Hb (OR = 0.921, 95% CI 0.892–0.951, *p* = <0.001), CRP (OR = 2.894, 95% CI 1.979–4.233, *p* = 0.00056), and SAS score (OR = 1.086, 95% CI 1.053 ~ 1.120, *p* = 0.00012) were independent risk factors for the development of nutritional impairment in elderly CHF inpatients, as shown in Table 4. The linearity of the logit for continuous variables (Hb, CRP, and SAS score) was assessed using the Box-Tidwell test, which showed no significant violation of the linear logit assumption (*p* = 0.382 for CRP, *p* = 0.451 for Hb, and *p* = 0.297 for SAS score). The final model retained the same five independent variables as the original analysis, with consistent ORs and significance levels, confirming the robustness of our findings.

**Table 4:** Multivariate logistic regression analysis of factors independently associated with nutritional impairment in elderly hospitalized patients with CHF.

Influencing factor	*Β* value	SE	Wald *X^2^*	*p*	*OR* value	*OR* value 95%*CI*
Smoking history (Yes vs. No)	0.891	0.303	8.650	0.003	2.437	1.346 ~ 4.414
NYHA classification (III-IV vs. I-II)	0.968	0.313	9.542	0.002	2.632	1.424 ~ 4.865
Hb (per 1 g/L increase)	−0.082	0.016	26.113	<0.001	0.921	0.892 ~ 0.951
CRP (per 1 mg/L increase)	1.063	0.194	30.046	<0.001	2.894	1.979 ~ 4.233
SAS score (per 1-point increase)	0.082	0.016	27.789	<0.001	1.086	1.053 ~ 1.120

### 3.4 Risk prediction model construction and validation

Based on the results of multifactorial logistic regression analysis plotted ROC smoking history, NYHA grading, Hb, CRP, SAS scores of the five independent risk factors to construct a column line graph model in order to visualize and individually assess the probability of the occurrence of malnutrition in patients hospitalized with CHF, the total sum of the prediction point scores corresponding to each variable corresponds to the bottom scale of the column line graph, see Figure 3; In the nomogram, variable coding is as follows: smoking history 0=No,1=Yes , NYHA classification 0=I−II,1=III−IV ; Hb, CRP, and SAS scores are entered as continuous variables with their original units (g/L, mg/L, and points, respectively). The total points correspond to the predicted probability of nutritional impairment on the bottom scale. Using the Hosmer and Lemeshow goodness of fit (GOF) test method was used to assess the model fit showed that its *p*-value was: 0.266, statistic was: 9.981, ROC curve was used for apparent performance evaluation, and the results showed that the apparent AUC value was 0.869, 95% CI was (0.814 ~ 0.923), indicating that the overall prediction model accuracy is high, see Figure 4. After internal validation using bootstrap resampling with 1,000 repetitions, the optimism-corrected AUC was 0.837 (95% CI: 0.778–0.891), indicating that the model’s discriminative ability remained satisfactory after adjusting for overfitting. The Brier score was 0.850 (Figure 5). The calibration curve showed good agreement between predicted and observed probabilities. The calibration slope was 0.917 (95% CI: 0.823–1.011), and the calibration intercept was −0.052 (95% CI: −0.214 to 0.110), indicating no significant miscalibration (calibration slope close to 1 and intercept close to 0).

**Figure 3:**
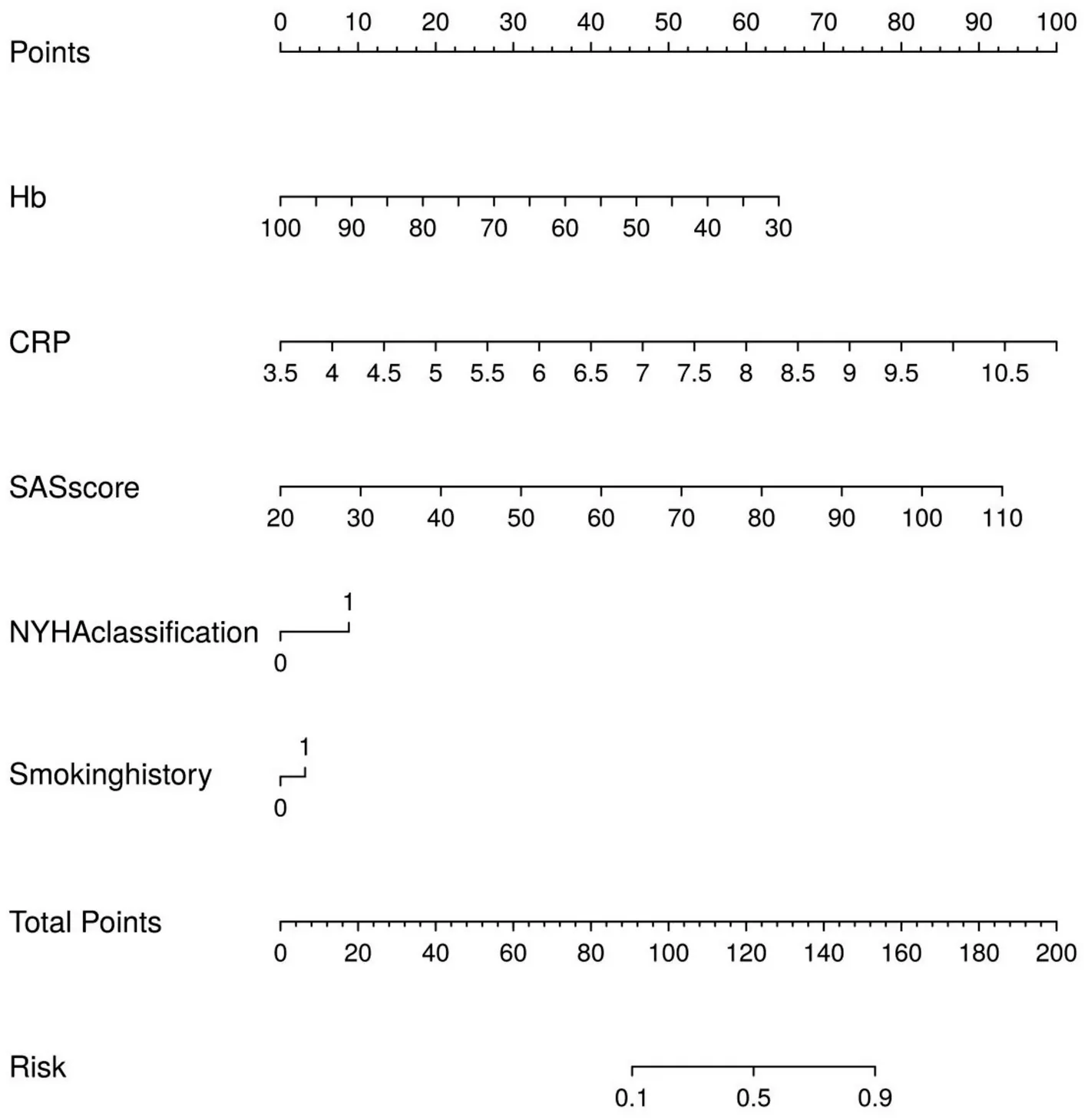
Construction of nomogram risk prediction model. Variable coding: Smoking history (0 = No, 1 = Yes); NYHA classification (0 = I-II, 1 = III-IV); Hb (g/L), CRP (mg/L), and SAS score (points) are entered as continuous variables.

**Figure 4:**
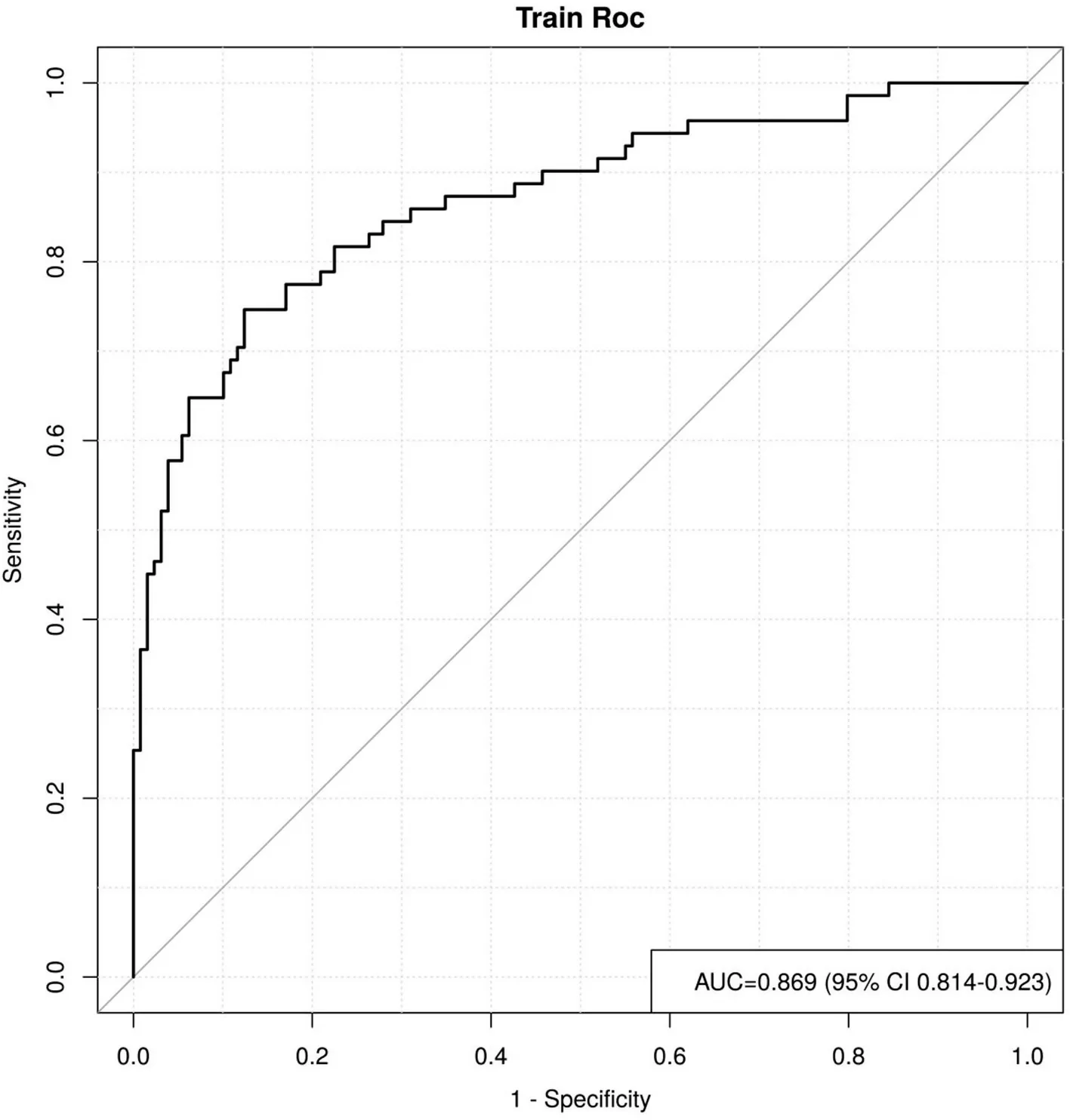
ROC curve (apparent performance, *n* = 200).

**Figure 5:**
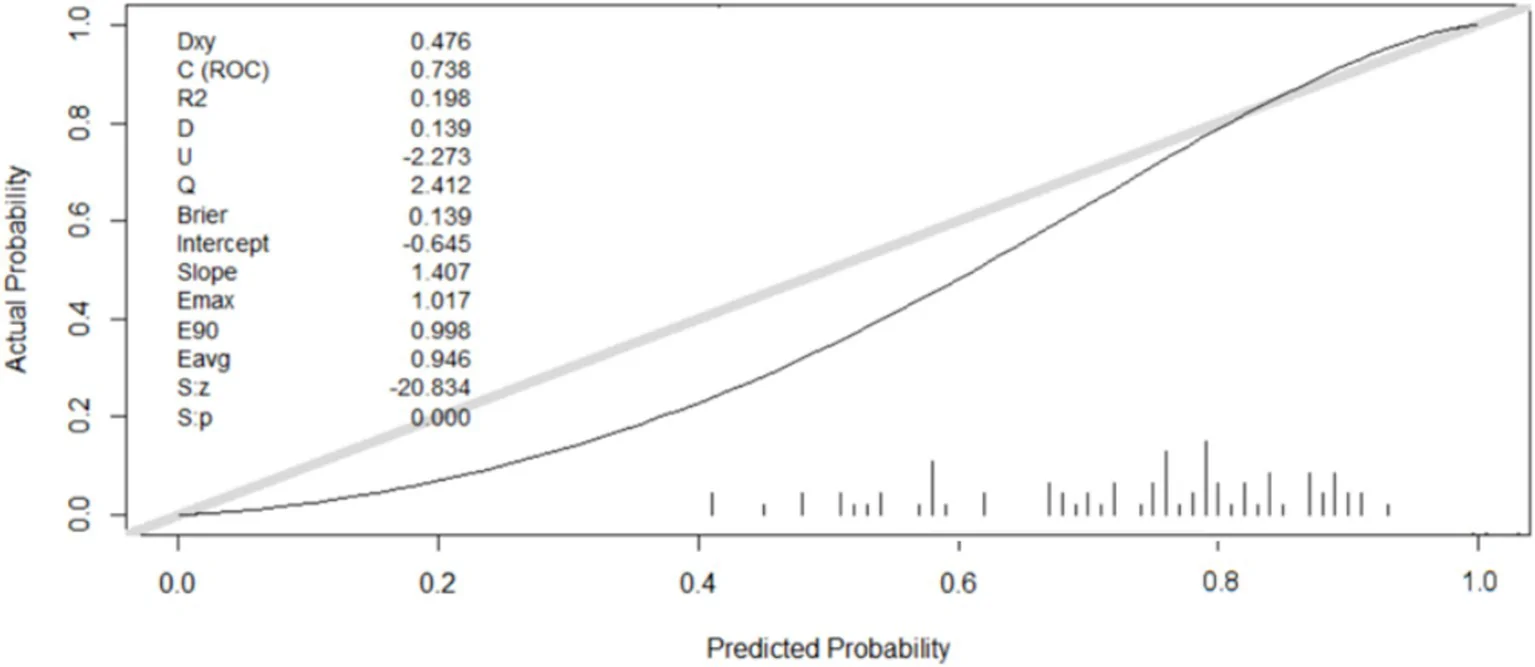
Brier score.

### 3.5 Overlap between GNRI and NRS2002 screening results

To provide full transparency regarding the combined screening algorithm, the numbers of patients identified by each tool individually and their overlap are presented in Table 1. Among the 200 enrolled patients, GNRI ≤98 identified 89 patients (44.5%) at nutritional risk, while NRS2002 ≥ 3 identified 78 patients (39.0%). The combined “and” algorithm identified 71 patients (35.5%) who were positive by both tools, which constituted the nutritional impairment group for subsequent analyses. The overlap between the two screening tools is visualized in a Venn diagram (Figure 2).

### 3.6 Comparison of NYHA classification and incidence of malnutrition before and after the implementation of diversified management model

In this uncontrolled before-after exploratory analysis of the same 200 patients, paired admission and discharge data were compared using McNemar’s test for paired categorical variables. The rates of patients with NYHA classification of grade I (27.50% (55/200) vs. 17.00% (34/200)) and grade II (57.00% (114/200) vs. 50.50% (101/200)) were higher than those of patients before the implementation, and patients with NYHA classification of grade III (10.00% (20/200) vs. 22.50% (45/200)), grade IV (5.50% (11/200) vs. 10.00% (20/200)) and the incidence of malnutrition (16.00% (32/200) vs. 35.50% (71/200)), McNemar’s test showed statistically significant changes in NYHA classification (dichotomized as I-II vs. III-IV: McNemar *χ*^2^ = 12.07, *p* < 0.001) and nutritional impairment status (McNemar *χ*^2^ = 17.36, *p* < 0.001), thus indicating that in this exploratory uncontrolled before-after comparison, the diversified management mode can effectively improve patients’ cardiac function and nutritional status, see Table 5.

**Table 5:** NYHA classification and incidence of malnutrition before and after the implementation of diversified management model.

Argument		Before implementation (*n* = 200)	Post-implementation (*n* = 200)	McNemar *x^2^*	*p*
NYHA classification	I Level	34 (17.00)	55 (27.50)	19.691	<0.001
	II Level	101 (50.50)	114 (57.00)		
	III Level	45 (22.50)	20 (10.00)		
	IV Level	20 (10.00)	11 (5.50)		
Incidence of malnutrition		71 (35.50)	32 (16.00)	19.888	<0.001

### 3.7 Uncontrolled before-after exploratory analysis: comparison of Hb, CRP and SAS scores before and after the implementation of diversified management mode

In this uncontrolled before-after exploratory analysis of the same 200 patients, paired admission and discharge data were compared using paired *t*-tests for paired continuous variables. After the implementation of the patient’s Hb (69.02 ± 8.98 vs. 62.41 ± 10.26) g/L, CRP (5.12 ± 0.74 vs. 5.90 ± 0.88) mg/L, and SAS scores (50.21 ± 8.46 vs. 56.25 ± 11.34) were compared with those before implementation. The difference was statistically significant (*p* < 0.05) when compared with the pre-implementation period, thus indicating that in this exploratory uncontrolled before-after comparison, see Figure 6 (*n* = 200 per group; data are presented as mean ± SD; *p* < 0.05 for all comparisons by paired *t*-test).

**Figure 6:**
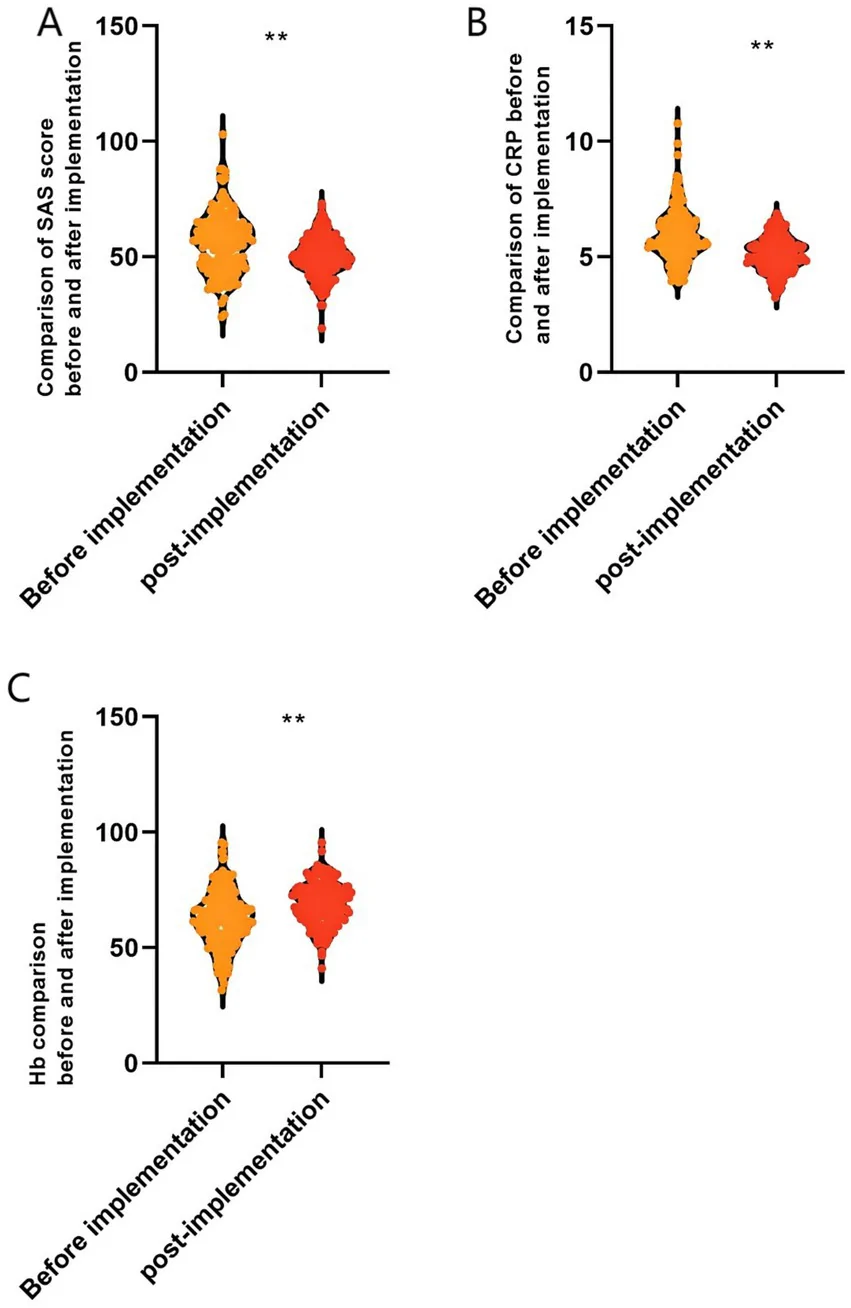
Comparison of Hb, CRP, and SAS scores before and after the implementation of diversified management mode (*n* = 200 per group; data are presented as mean ± SD; *p* < 0.05 by paired *t*-test for all comparisons). Error bars represent standard deviation.

## 4 Discussion

According to statistics, China currently has about 4 million CHF patients among adults aged 35 to 74 years. In the southern region, with increasing age, the prevalence of heart failure CHF will also rise significantly; patients aged 60 to 70 years accounted for about 39.2%, and those aged 70 to 80 years old accounted for about 60.8%; the difference is statistically significant (21). According to the relevant research data, CHF exhaustion patients are often nutritionally impaired due to systemic metabolic changes and increased body consumption, with an incidence of 16 to 62%, reducing the survival rate of patients and increasing the risk of readmission due to deterioration of the condition (22). Therefore, actively analyzing the relevant factors associated with the occurrence of malnutrition in patients is the key to taking relevant measures to prevent and control it.

Through the data of this study, the comparison of smoking history, NYHA classification, hemoglobin (Hb), C-reactive protein (CRP), and Self-Assessment Scale of Anxiety (SAS) scores in the impairment group and the non-impairment group showed that the difference was statistically significant (*p* < 0.05); all of the above mentioned indexes were independently associated with the development of nutritional impairment in elderly CHF inpatients (*p* < 0.05). It is important to note that, given the observational cross-sectional design of this risk factor analysis, these associations should not be interpreted as causal relationships. We cannot determine whether low Hb, elevated CRP, and high anxiety scores are causes, consequences, or simply indicators of greater overall disease severity that coexists with malnutrition in this population. Regarding the hemoglobin levels observed in our cohort, the mean admission Hb of 56.12 g/L in the impairment group and 65.88 g/L in the non-impairment group indicate very severe anemia, which is substantially lower than typical values reported in general CHF populations. However, these values are consistent with previous studies of advanced CHF patients with concomitant nutritional impairment, where anemia of chronic disease, iron deficiency, and malnutrition collectively contribute to profound hemoglobin reduction. Notably, our laboratory uses the Sebia automated agarose gel electrophoresis system with stringent quality control, and all measurements were performed on admission fasting samples prior to any transfusion intervention. Patients with Hb < 60 g/L received packed red blood cell transfusions and/or intravenous iron supplementation per guideline-directed therapy during hospitalization, which was consistently applied across both groups. Corsetti et al. (23) reported that a history of smoking was associated with an increased risk of nutritional impairment in CHF patients. Smoking interferes with metabolism and oxidative stress while suppressing appetite and increasing body energy expenditure. On the one hand, nicotine and carbon monoxide in tobacco can lead to vasoconstriction, reduce gastrointestinal blood flow, decrease the efficiency of nutrient absorption, and induce oxidative stress that can damage the barrier function of the intestinal mucosa and exacerbate the increase in intestinal permeability, affecting the absorption of proteins and micronutrients; on the other hand, nicotine inhibits the absorption of proteins and micronutrients through its effects on the central nervous system. Additionally, nicotine suppresses appetite by acting on the central nervous system (e.g., hypothalamus), while simultaneously increasing the body’s resting metabolic rate. This results in energy expenditure surpassing intake, which accelerates muscle catabolism and heightens the risk of nutritional impairment. Previous studies have indicated that the NYHA classification is a cardiac function grading scheme proposed by the New York Heart Association in the United States. It is primarily utilized to assess cardiac function in patients with heart failure and is graded based on patients’ activity tolerance and symptomatic manifestations. Higher grades correspond to poorer cardiac function within an individual (16, 24). The proportion of elderly CHF inpatients classified as NYHA grade III-IV in the impairment group was significantly higher than that in the non-impairment group. This finding indicates that the overall cardiac function status of patients in the impairment group is poorer, leading to stagnation of systemic circulation and increased intestinal venous pressure. Consequently, this triggers mucosal edema and an imbalance in intestinal bacterial flora, which decreases digestive enzyme activity and nutrient absorption. Additionally, symptoms such as dyspnea and sedentary respiration result in increased energy expenditure during eating. Patients experience systemic tissue hypoxia due to inefficient cardiac pumping, which leads to heightened anaerobic metabolism and lactic acid accumulation. This further suppresses appetite, resulting in systemic metabolic alterations, reduced muscle mass and function, ultimately contributing to nutritional impairment within the organism, which is in line with the arguments of the previous study (37). Hb is a special protein that transports oxygen in erythrocytes. It is a protein that makes the blood red, and it is made up of beads and hemoglobin, whose beads are composed of two different pairs of beads. Portion is a tetramer composed of two different pairs of beadin chains (*α* and *β* chains), and its level is closely related to nutritional status (25, 26). The Hb level of the impairment group was significantly lower than that of the non-impairment group, suggesting that the oxygen-carrying capacity of the patients in the non-impairment group decreased. In the setting of CHF, lower Hb may trigger a compensatory increase in cardiac output, potentially further aggravating the burden on the heart, which has been described as a potential “anemia-heart failure” cycle. In addition, tissue hypoxia may activate hypoxia-inducible factors (HIFs), which have been implicated in promoting muscle protein catabolism, inhibiting the mTOR pathway, and reducing protein synthesis. Previous studies have demonstrated that CRP is a protein that significantly elevates in plasma during infection or tissue damage. It activates complement pathways and enhances phagocytosis while playing a regulatory role by eliminating pathogenic microorganisms that invade the organism as well as damaged, necrotic, and apoptotic cells. Consequently, CRP plays an essential protective role in the body’s natural immune response. Its association with the nutritional status of the organism is mediated through inflammatory responses (27). CHF is regarded as a systemic inflammatory disease, and this inflammatory state is similar to that of tumors and autoimmune diseases. CRP, as an inflammatory marker, can be rapidly increased up to a thousand-fold when inflammation occurs in the organism. Experimental studies suggest that CRP may activate the ubiquitin-proteasome system through the NF-κB pathway and potentially accelerate muscle proteolysis (e.g., myosin light chain), leading to decreased glucose utilization, increased lipolysis, and intestinal barrier function disruption, which may not only increase body energy expenditure but also act directly on the hypothalamic arcuate nucleus to inhibit neuropeptide Y secretion, while stimulating pro-inflammatory signaling (e.g., POMC neurons), potentially triggering anorexia and early satiety. The SAS was compiled by W. K. Zung in 1971 to assess the subjective feeling of anxiety in patients and its change in treatment, and it can be useful in the treatment of CHF when the elderly patient is are physiologically unwell and facing the pressure of prolonged treatment, they often show symptoms of anxiety such as nervousness, worry, fear and anxiety, as well as sweating, flushing and dry mouth (28). Referring to the study of Sánchez-Torralvo et al. (29), it can be observed that, on one hand, anxiety induces neuroendocrine disorders by activating the sympathetic-adrenomedullary and hypothalamic–pituitary–adrenal axes. This activation leads to an increase in the secretion of catecholamines and cortisol, promotes gluconeogenesis and lipolysis, inhibits protein synthesis, and accelerates muscle wasting. On the other hand, anxiety adversely impacts patients’ behavior, often manifesting through symptoms such as sweating, flushing, and dry mouth. Furthermore, anxiety negatively influences patients’ eating habits by resulting in decreased appetite, irregular meal patterns, and selective dietary choices (e.g., high-sugar and low-protein diets). These factors contribute to insufficient nutrient intake. Coupled with poor sleep quality that elevates resting energy expenditure, this ultimately results in a scenario characterized by low nutrient intake alongside high demand and reduced absorptive capacity—thereby increasing the risk of nutritional impairment. In this study, a nomogram prediction model was developed by integrating various factors. The complex multifactorial regression equations were transformed into graphical representations, enhancing the visualization and readability of abstract data outcomes. The approximate probability of nutritional impairment occurrence in patients with congestive heart failure (CHF) was assessed using different predictor variables. Receiver Operating Characteristic (ROC) curves were generated for internal validation, revealing an Area Under the Curve (AUC) value of 0.869, with a 95% Confidence Interval (CI) ranging from 0.814 to 0.923. The model fit yielded a *P*-value of 0.266 and a statistic of 9.981, indicating that the difference between predicted probabilities and actual probabilities was not statistically significant. The model demonstrated good discriminative ability with an apparent AUC of 0.869 and an optimism-corrected AUC of 0.837 after bootstrap internal validation. Furthermore, the Brier score of 0.139 indicated that the predicted probabilities were reasonably well-calibrated, and the calibration slope of 0.917 with an intercept of −0.052 confirmed no significant miscalibration. The Hosmer-Lemeshow test also supported good model fit (*p* = 0.266). However, it must be emphasized that the apparent AUC of 0.869 represents the model’s performance in the same development sample and is therefore optimistic. The optimism-corrected AUC of 0.837 provides a more realistic estimate of the model’s discriminative ability. Overall, these multiple performance metrics consistently suggest that the nomogram has satisfactory predictive accuracy, although external validation is still needed before clinical implementation. These findings suggest that the predictive model may offer substantial clinical support by demonstrating how these risk factors may interact through metabolic, inflammatory, neuroendocrine, and other multidimensional pathways that may contribute to nutritional impairment. Consequently, it is imperative for clinics to provide optimal support through nutritional assistance, psychological interventions, cardiac function rehabilitation exercises, and other multi-targeted integrated management strategies in order to mitigate this vicious cycle slightly and improve clinical prognosis.

It is important to acknowledge several potential biases that may affect the interpretation of our findings. First, reverse causality cannot be excluded: malnutrition itself may lead to lower Hb, elevated inflammatory markers, and worse anxiety symptoms, rather than the reverse. Second, residual confounding may persist despite multivariate adjustment, as unmeasured variables such as renal function, detailed medication profiles, and socioeconomic status were not included in the analysis. Third, outcome misclassification may have occurred because nutritional impairment was defined using screening tools (GNRI and NRS2002) rather than a formal diagnostic framework such as GLIM, and both GNRI components (albumin and body weight) can be influenced by fluid status in CHF patients. Fourth, selection bias may be present due to the single-centre retrospective design and the exclusion of patients with incomplete medical records. Fifth, the apparent performance of the prediction model (AUC 0.869) is optimistic, and the optimism-corrected AUC of 0.837 provides a more realistic estimate.

It is important to acknowledge that GNRI, the tool used to define nutritional impairment in this study, incorporates both serum albumin and body weight in its calculation. In patients with CHF, several factors may influence these components independently of true nutritional status. Specifically, hemodilution from fluid overload and congestion can lower serum albumin levels without reflecting protein-energy malnutrition, and edema can increase body weight, thereby paradoxically elevating GNRI values and potentially underestimating nutritional risk in volume-overloaded patients. Conversely, diuretic therapy may reduce edema and body weight, which could affect GNRI classification. Therefore, the observed associations between NYHA classification, Hb, CRP, and nutritional impairment may be partly mediated by disease severity and inflammation rather than representing purely independent causal relationships. For example, more advanced NYHA class is associated with greater fluid retention and congestion, which directly affects both albumin and weight; higher CRP reflects systemic inflammation that suppresses albumin synthesis and accelerates muscle catabolism; and lower Hb in CHF results from a combination of hemodilution, impaired erythropoiesis, and chronic inflammation. These complex interrelationships should be considered when interpreting our findings. We acknowledge that variable selection based solely on *p*-values is insufficient; instead, our selection was guided by clinical relevance and prior literature, and we recommend that future studies employ causal diagrams (directed acyclic graphs) and more advanced methodologies to better disentangle the direct and indirect effects of these variables on nutritional impairment in CHF.

Diversified nursing interventions focus on reflecting the concept of “person-centered” care, holistic care through multiple perspectives, reflecting the nursing perspective of “promoting health and alleviating pain” in all aspects, and the intervention program in the standardized, scientific nursing procedures to achieve, so it can be The intervention program is achieved under standardized and scientific nursing procedures, so it is possible to obtain better nursing care (30). In this uncontrolled before-after exploratory analysis, the data of this study showed that after the implementation of the diversified management model, the patients’ NYHA classification, nutritional impairment incidence, Hb, CRP, and SAS scores were significantly improved compared with those before the implementation of the diversified management model (*p* < 0.05). However, as this was an exploratory analysis without a control group, these findings should be interpreted with caution and considered hypothesis-generating rather than confirmatory. This is mainly due to the diversified nursing intervention in the pre-discharge according to the patient’s gender, age, body mass, height, usual dietary habits, economic status, based on the patient’s actual nursing needs to develop targeted, individualized dietary programs, if the patient has a poor dietary structure and dietary habits, the nursing staff should be the patient’s nutritional guidance, so as to improve the reasonable dietary structure, and at the same time in the post-discharge to the patient’s unsuitable Dietary structure and poor dietary habits of patients are effectively corrected, patients are informed of the importance of small meals, light diet for nursing treatment, close attention to the patient’s diet, from the direction of physiological-psychological two-way regulation, by improving the patient’s protein-energy balance, enhance immune function and recovery speed, to create a good physiological basis for psychological interventions; psychological interventions (e.g., cognitive-behavioral therapy) through the alleviation of anxiety and depression, enhance patient acceptance of the nutritional program. Psychological interventions (e.g., cognitive-behavioral-therapy) enhance patients’ acceptance of nutritional regimens by alleviating anxiety and depression, forming a positive cycle. Personalized nutritional regimens (e.g., high-protein diets), together with psychological counseling, help patients understand the need for treatment and reduce dietary interruptions due to cognitive bias. Regular psychological assessment can identify mood disorders associated with abnormal nutrition and metabolism (such as eating disorders), and timely adjust intervention strategies to improve nutritional status and psychological state, which is in line with the argument of International EM et al. (31). reference to previous studies, it can be seen that the aging of functional organs and the change of physiological environment of elderly CHF inpatients make their adaptive ability to life weakened, and if symptoms such as respiratory failure occur, the patient’s body and mind will be extremely painful, which is easy to cause anxiety, depression and other adverse emotions, and if the patient is in anxiety, panic, sadness, pain and other adverse emotions, a series of stress and inflammatory reactions will occur (32). Clinical scholars frequently apply this concept in nutritional impairment nursing interventions. Nursing staff conduct comprehensive assessments of patients’ disease progression and physical status, implementing physiological, psychological, and dietary care measures to foster a harmonious patient-doctor relationship and enhance treatment efficiency. In this study, the implementation of a diversified management model through targeted rehabilitation exercises is explored. A remote monitoring system utilizing wearable devices enables real-time tracking of heart rate, blood oxygen levels, and other vital indicators. This approach can increase myocardial blood supply, promote the formation and development of collateral circulation within the myocardium, significantly improve lipid metabolism in the body, reduce susceptibility factors’ threats, enhance psycho-emotional recovery rates, improve both quantity and quality of mitochondria present in muscle tissue, elevate intramuscular antioxidant enzyme content, and ultimately improve cardiac function grades. Additionally, in conjunction with assessments conducted by rehabilitation specialists regarding exercise tolerance levels, training intensity was dynamically adjusted to mitigate the risk of readmission due to overexertion. Simultaneously, nursing staff analyzed successful cases from previous treatments at the hospital through meticulous refinement and analysis alongside health education initiatives and psychological care interventions aimed at enhancing patients’ understanding of their prognostic rehabilitation programs—thereby improving their overall treatment engagement. At the same time, nursing staff improve patients’ knowledge of the prognostic rehabilitation program through detailed analysis of the hospital’s previous treatment successes, health education and psychological care interventions, which in turn improve their treatment compliance and cooperation, and eliminate the generation of a negative mindset from the source, in order to help patients establish confidence in restoring their health, gradually alleviate patients’ negative emotions, such as anxiety and depression, and reduce the psychological burden on patients; through the combination of nutritional support and psychological interventions, patients’ compliance with feeding is improved, and the linkage of rehabilitation training and remote monitoring reduces the risk of readmission, promotes the rapid recovery of the organism, and improves the patients’ nutritional state, cardiac function and Adverse emotions.

However, it is critical to emphasize that the before-after comparison in this study was conducted without a parallel control group. Therefore, it is not possible to attribute the observed improvements solely to the diversified management model. Several alternative explanations must be considered. First, all patients received standard guideline-directed CHF medical therapy (including diuretics, ACE inhibitors/ARBs, beta-blockers, and other agents) throughout hospitalization, and improvements in cardiac function and inflammatory markers may partially reflect the natural response to optimized medical management rather than the nursing intervention per se. Second, the hospitalization period itself provides a controlled environment with regular meals, medication adherence, and close monitoring, which may contribute to clinical improvement regardless of the specific diversified program—a phenomenon known as the “hospital effect” or regression to the mean. Third, the timing of outcome measurement (admission vs. discharge) may coincide with the natural trajectory of acute decompensation recovery, where patients typically show improvement over time. Fourth, without a control group, we cannot exclude the possibility that selection bias or confounding by unmeasured variables (e.g., differential length of stay, severity of illness at admission, or socioeconomic factors) influenced the observed changes. Finally, the lack of long-term follow-up means we cannot determine whether these improvements were sustained after discharge or whether they translate into meaningful clinical outcomes such as reduced readmission or mortality. Therefore, our findings should be interpreted as preliminary observational evidence of within-cohort changes rather than as proof of the intervention’s causal efficacy. Rigorous randomized controlled trials or well-designed quasi-experimental studies with control groups are urgently needed to confirm whether the diversified management model independently improves clinical outcomes in elderly CHF patients.

The shortcomings of this study: (1) this is a single-centre retrospective observational study with a relatively small sample size of 200 patients. The single-centre design limits the generalizability of our findings, as the study population was drawn from a specific geographic region with potentially homogeneous ethnicity, cultural background, and socioeconomic status, which may not be representative of broader CHF populations. The sample size, while meeting the minimum EPV requirement of 10 for the logistic regression model, is still modest and may limit the precision of effect estimates and the ability to detect smaller but clinically meaningful associations. Furthermore, the retrospective data collection introduces inherent risks of selection bias, as we could only include patients with complete medical records, potentially excluding those with missing data who might differ systematically from the included cohort. Additionally, a small sample size relative to the number of independent variables may lead to overfitting, as reflected by the modest decrease from apparent AUC of 0.869 to optimism-corrected AUC of 0.837; (2) although we used forward stepwise selection including all clinically relevant variables to minimize selection bias, stepwise regression has inherent limitations such as dependence on the order of variable entry and potential overfitting; future studies should employ more rigorous methods such as LASSO regression or include all *a priori* clinically relevant covariates to further validate these findings; (3) the nomogram prediction model has undergone internal validation using bootstrap resampling; however, independent external validation in an independent cohort has not yet been performed due to the single-centre retrospective design and limited sample size. Therefore, the model should currently be considered as an auxiliary screening tool rather than a definitive clinical decision-making instrument, and we strongly recommend that future multicenter studies with larger sample sizes be conducted to externally validate and refine the model before its widespread clinical application; (4) importantly, the before-after comparison of the multicomponent management program was conducted without a parallel control group. This design limitation precludes any causal attribution of the observed improvements to the intervention itself. The improvements in NYHA classification, nutritional status, inflammatory markers, and anxiety observed at discharge may be confounded by concurrent standard CHF medical therapy, the natural history of acute decompensation recovery, regression to the mean, the “hospital effect” of a controlled inpatient environment, variation in length of stay, and unmeasured selection bias. Without a control group, we cannot differentiate the specific effects of the diversified management model from these confounding factors. Additionally, since the intervention was applied to all patients universally without a comparator group receiving usual care without the program, we cannot determine the incremental benefit of the diversified management beyond standard medical therapy; (5) despite multivariate adjustment for a wide range of clinically relevant variables, residual confounding may still persist due to unmeasured factors such as detailed medication profiles (e.g., doses and adherence), renal function, socioeconomic status, educational level, social support systems, and dietary habits outside the hospital setting. These unmeasured confounders could potentially influence both the exposure and outcome, leading to biased effect estimates; (6) the absence of post-discharge follow-up means the sustainability of the observed inpatient improvements and their impact on long-term outcomes such as readmission and mortality remain unknown. We cannot determine whether the improvements in cardiac function, nutritional status, inflammatory markers, and anxiety observed at discharge are maintained after patients return to their home environment; (7) additionally, as GNRI and NRS2002 are screening tools rather than diagnostic instruments, future studies should consider confirming nutritional impairment using the GLIM diagnostic framework to enhance clinical robustness. Furthermore, the extremely low Hb values observed in our cohort should be interpreted with caution; while they reflect the severe disease burden of this advanced CHF population, future multicenter studies with larger sample sizes are needed to validate these findings and explore the underlying mechanisms of profound anemia in nutritionally impaired elderly CHF patients; (8) the cross-sectional nature of the risk factor analysis precludes any conclusions about temporal or causal relationships between the identified factors and nutritional impairment. We can only report associations, which may be bidirectional or confounded by disease severity. We will increase the sample size and set up a control group in future research to obtain convincing results. In order to conduct a comprehensive study on the diversified management model of nursing, possible confounding factors and deviations, such as socio-economic factors and comorbidities, should be controlled in future research. Long-term post-discharge follow-up should also be incorporated to evaluate the durability of the intervention effects. In our future research, we will try our best to contact other hospitals to conduct multi-center and multi-sample studies, long-term follow-up of patients, reduce sampling errors and selective bias, and obtain more accurate results.

## 5 Conclusion

In this single-center retrospective analysis, smoking history, advanced NYHA class, low hemoglobin level, elevated CRP, and high anxiety score were found to be independently associated with nutritional impairment in elderly hospitalized CHF patients. The nomogram constructed based on these five indicators may serve as an auxiliary tool for identifying high-risk patients in clinical settings. However, given the observational cross-sectional design, these associations should be interpreted as exploratory rather than causal, and the prediction model’s apparent performance (AUC 0.869) requires further validation in independent external cohorts before clinical implementation. The uncontrolled before-after exploratory analysis suggested within-cohort improvements in cardiac function, nutritional status, inflammatory markers, and anxiety following the multicomponent management program; nevertheless, these findings are preliminary and hypothesis-generating, as the absence of a control group precludes any causal attribution. Future prospective multicenter studies with larger sample sizes are needed to validate the predictive model, and well-designed randomized controlled trials are urgently required to confirm whether the diversified management approach independently improves clinical outcomes in this population.

## Data Availability

The original contributions presented in the study are included in the article/supplementary material, further inquiries can be directed to the corresponding author.
